# Supplementary material for: Update on the Prevalence, Incidence, Mortality, and Trends in Treatment of Inflammatory Bowel Disease in a Population-Based Registry in Catalonia Between 2017 and 2023
Source: J Clin Med. 2025 Aug 12;14(16):5711. doi: 10.3390/jcm14165711 (PMC12386801; doi:10.3390/jcm14165711)

SUPPLEMENTARY FILES

Supplementary Table S1. ICD-10 diagnoses for CD and UC

**K50**	**Crohn disease [regional enteritis]**
K50.0	Crohn disease of small intestine: Crohn disease [regional enteritis] of duodenum, ileum, jejunum, ileitis (regional, terminal).<br>* Exclude Crohn disease of large intestine (K50.8).
K50.1	Crohn disease of large intestine: Colitis (Granulomatous, regional). Crohn disease [regional enteritis] of colon, large bowel, rectum.<br>* Exclude Crohn disease of small intestine (K50.8).
K50.8	Other Crohn disease: Crohn disease of both small and large intestine
K50.9	Crohn disease, unspecified: Regional enteritis NOS
**K51**	**Ulcerative Colitis**
K51.0	Ulcerative (chronic) pancolitis. backwash ileitis
K51.2	Ulcerative (chronic) proctitis
K51.3	Ulcerative (chronic) rectosigmoiditis
K51.4	Inflammatory polyps
K51.5	Left sided colitis; left hemicolitis
K51.8	Other ulcerative colitis
K51.9	Ulcerative colitis, unspecified

Supplementary Table S2. List of drugs used in IBD and ATC codes.

**Immunosuppressant**	
L04AX01	Azathioprine
L01BB02	Mercaptopurine
L04AX03	Methotrexate
L04AD01	Cyclosporine
**Salicylates**	
A07EC02	Mesalazine
A07EC01	Sulfasalazine
**Biologic treatment**	
L04AB02	Infliximab
L04AB02	Infliximab biosimilar
L04AB04	Adalimumab
L04AB06	Golimumab
L04AA33	Vedolizumab
L04AC05	Ustekinumab
L04AF01	Tofacitinib
L04AF04	Filgotinib

L04AF03	Upadacitinib
**Systemic corticosteroids**	
H02AB07	Prednisone
H02AB06	Prednisolone
H02AB04	Methylprednisolone
H02AB09	Hydrocortisone
H02AB13	Deflazacort
H02AB08	Triamcinolone
H02AB02	Dexamethasone
H02AB08	Triamcinolone
H02AB01	Betamethasone
**Topical corticosteroids**	
A07EA07	Beclomethasone dipropionate
A07EA06	Budesonide
C05AA12	Triamcinolone diacetate
**Janus Kinase Inhibitors**	
L04AF04	Filgotinib
L04AF01	Tofacitinib

L04AF03	Upadacitinib

Supplementary Table S3. ICD-10 codes for surgical procedures in IBD.

CODE	DESCRIPTION
**Ostomies**	
0D1H0Z4	Cecum to cutaneous shunt, open approach
0D1H4Z4	Cecum to cutaneous shunt, percutaneous endoscopic approach
0D1H8Z4	Cecum to cutaneous shunt, natural or artificial orifice endoscopic approach
0D1K0Z4	Ascending colon to cutaneous shunt, open approach
0D1K4Z4	Ascending colon shunt to cutaneous, percutaneous endoscopic approach
0D1K8Z4	Ascending colon to cutaneous shunt, natural or artificial orifice endoscopic approach
0D1L0Z4	Transverse colon to cutaneous shunt, open approach
0D1L4Z4	Transverse colon to cutaneous shunt, percutaneous endoscopic approach
0D1L8Z4	Transverse colon to cutaneous shunt, natural or artificial orifice endoscopic approach
0D1N0Z4	Sigmoid colon to cutaneous shunt, open approach
0D1N4Z4	Sigmoid to cutaneous colon shunt, percutaneous endoscopic approach
0D1N8Z4	Sigmoid to cutaneous colon shunt, natural or artificial orifice endoscopic approach
0D1H0Z4	Cecum to cutaneous shunt, open approach
0D1H4Z4	Cecum to cutaneous shunt, percutaneous endoscopic approach
0D1H8Z4	Cecum to cutaneous shunt, natural or artificial orifice endoscopic approach
0D1K0Z4	Ascending colon to cutaneous shunt, open approach
0D1K4Z4	Ascending colon shunt to cutaneous, percutaneous endoscopic approach
0D1K8Z4	Ascending colon to cutaneous shunt, natural or artificial orifice endoscopic approach
0D1L0Z4	Transverse colon to cutaneous shunt, open approach
0D1L4Z4	Transverse colon to cutaneous shunt, percutaneous endoscopic approach
0D1L8Z4	Transverse colon to cutaneous shunt, natural or artificial orifice endoscopic approach
0D1N0Z4	Sigmoid colon to cutaneous shunt, open approach
0D1N4Z4	Sigmoid to cutaneous colon shunt, percutaneous endoscopic approach
0D1N8Z4	Sigmoid to cutaneous colon shunt, natural or artificial orifice endoscopic approach
0D1H0Z4	Cecum to cutaneous shunt, open approach
0D1H4Z4	Cecum to cutaneous shunt, percutaneous endoscopic approach
0D1H8Z4	Cecum to cutaneous shunt, natural or artificial orifice endoscopic approach
0D1K0Z4	Ascending colon to cutaneous shunt, open approach
0D1K4Z4	Ascending colon shunt to cutaneous, percutaneous endoscopic approach
0D1K8Z4	Ascending colon to cutaneous shunt, natural or artificial orifice endoscopic approach
0D1L0Z4	Transverse colon to cutaneous shunt, open approach
0D1L4Z4	Transverse colon to cutaneous shunt, percutaneous endoscopic approach

0D1L8Z4	Transverse colon to cutaneous shunt, natural or artificial orifice endoscopic approach
0D1N0Z4	Sigmoid colon to cutaneous shunt, open approach
0D1N4Z4	Sigmoid to cutaneous colon shunt, percutaneous endoscopic approach
0D1N8Z4	Sigmoid to cutaneous colon shunt, natural or artificial orifice endoscopic approach
0H87XZZ	Skin division, abdomen, external approach
0D1B0Z4	Ileum-to-cutaneous shunt, open approach
0D1B4Z4	Ileum shunt to cutaneous, percutaneous endoscopic approach
0D1B8Z4	Ileum to cutaneous bypass, natural or artificial orifice endoscopic approach
0D1B0Z4	Ileum-to-cutaneous shunt, open approach
0D1B4Z4	Ileum shunt to cutaneous, percutaneous endoscopic approach
0D1B8Z4	Ileum to cutaneous bypass, natural or artificial orifice endoscopic approach
0D1B0Z4	Ileum-to-cutaneous shunt, open approach
0D1B4Z4	Ileum shunt to cutaneous, percutaneous endoscopic approach
0D1B8Z4	Ileum to cutaneous bypass, natural or artificial orifice endoscopic approach
0D1B0Z4	Ileum-to-cutaneous shunt, open approach
0D1B4Z4	Ileum shunt to cutaneous, percutaneous endoscopic approach
0D1B8Z4	Ileum to cutaneous bypass, natural or artificial orifice endoscopic approach
0H87XZZ	Skin division, abdomen, external approach
0H87XZZ	Skin division, abdomen, external approach
0WBFXZ2	Abdominal wall excision, external approach, stoma
0WQFXZ2	Abdominal wall repair of stoma, external approach
0WBFXZ2	Abdominal wall excision, external approach, stoma
0WQFXZ2	Abdominal wall repair of stoma, external approach
0WQFXZ2	Abdominal wall repair of stoma, external approach
0WBFXZ2	Excision in abdominal wall, external approach, stoma
0WQFXZ2	Abdominal wall repair of stoma, external approach
0DQ80ZZ	Small bowel repair, open approach
0DQ90ZZ	Duodenal repair, open approach
0DQA0ZZ	Repair in jejunum, open approach
0DQB0ZZ	Repair in ileum, open approach
0DQE0ZZ	Large bowel repair, open approach
0DQF0ZZ	Large bowel repair, right, open approach
0DQG0ZZ	Large bowel repair, left, open approach
0DQH0ZZ	Blind repair, open approach
0DQK0ZZ	Ascending colon repair, open approach
0DQL0ZZ	Transverse colon repair, open approach
0DQM0ZZ	Descending colon repair, open approach
0DQN0ZZ	Sigmoid colon repair, open approach
0WQFXZ2	Abdominal wall repair of stoma, external approach
0DQ80ZZ	Small bowel repair, open approach
0DQ90ZZ	Duodenal repair, open approach
0DQA0ZZ	Repair in jejunum, open approach

0DQB0ZZ	Repair in ileum, open approach
0WQFXZ2	Abdominal wall repair of stoma, external approach
0DQE0ZZ	Large bowel repair, open approach
0DQH0ZZ	Blind repair, open approach
0DQN0ZZ	Sigmoid colon repair, open approach
0WQFXZ2	Abdominal wall repair of stoma, external approach
**Bowel resection**	
0DC80ZZ	Small bowel resection, open approach
0DCB0ZZ	Excision in ileum, open approach
0DCC0ZZ	Removal at ileocaecal valve, open approach
0DCE0ZZ	Removal in large intestine, open approach
0DCF0ZZ	Removal in large intestine, right, open approach
0DCG0ZZ	Removal in large intestine, left, open approach
0DCH0ZZ	Cecal excision, open approach
0DCK0ZZ	Removal in ascending colon, open approach
0DCL0ZZ	Excision in transverse colon, open approach
0DCM0ZZ	Removal in descending colon, open approach
0DCN0ZZ	Excision in sigmoid colon, open approach
0DB80ZZ	Small bowel excision, open approach
0DBB0ZZ	Excision in ileum, open approach
0DBC0ZZ	Ileocecal valve excision, open approach
0DBE0ZZ	Large bowel excision, open approach
0DBF0ZZ	Large bowel excision, right, open approach
0DBG0ZZ	Large bowel excision, left, open approach
0DBH0ZZ	Excision in the cecum, open approach
0DBK0ZZ	Excision in ascending colon, open approach
0DBL0ZZ	Excision in transverse colon, open approach
0DBM0ZZ	Excision in descending colon, open approach
0DBN0ZZ	Excision in sigmoid colon, open approach
0DB80ZZ	Small bowel excision, open approach
0DB80ZZ	Small bowel excision, open approach
0DB88ZZ	Small bowel excision, natural or artificial orifice endoscopic approach
0DBB0ZZ	Ileum excision, open approach
0DBC0ZZ	Ileocaecal valve excision, open approach
0DBE0ZZ	Large bowel excision, open approach
0DBF0ZZ	Large bowel excision, right, open approach
0DBG0ZZ	Large bowel excision, left, open approach
0DBH0ZZ	Cecum excision, open approach
0DBK0ZZ	Excision in ascending colon, open approach
0DBL0ZZ	Excision in transverse colon, open approach
0DBM0ZZ	Excision in descending colon, open approach

0DBN0ZZ	Excision in sigmoid colon, open approach
0DCJ0ZZ	Removal in the appendix, open approach
0DCP0ZZ	Excision in rectum, open approach
0DBP0ZX	Rectal excision, open approach, diagnostic
0DBP0ZZ	Rectal excision, open approach
**Other therapeutic procedures**	
0D1B07B	Ileum to ileum bypass with autologous tissue substitute, open approach
0D1B0JB	Ileum to ileum bypass with synthetic substitute, open approach
0D1B0KB	Ileum to ileum bypass with non-autologous tissue substitute, open approach
0D1B0ZB	Ileum tobileum bypass, open approach
0D1B47B	Ileum to ileum bypass with autologous tissue substitute, percutaneous endoscopic approach
0D1B4JB	Ileum to ileum bypass with synthetic substitute, percutaneous endoscopic approach
0D1B4KB	Ileum to ileum bypass with non-autologous tissue substitute, percutaneous endoscopic approach
0D1B4ZB	Ileum to ileum bypass, percutaneous endoscopic approach
0D1B87B	Ileum to ileum bypass with autologous tissue substitute, natural orifice or endoscopic artificial orifice approach
0D1B8JB	Ileum to ileum bypass with synthetic substitute, natural orifice or endoscopic artificial orifice approach
0D1B8KB	Ileum to ileum bypass with non-autologous tissue substitute, natural orifice or endoscopic artificial orifice approach
0D1B8ZB	Ileum to ileum bypass, natural orifice or endoscopic artificial orifice approach
0D1B8ZH	Ileum to cecum bypass, natural orifice or endoscopic artificial orifice approach
0D1B07P	Ileum to rectum bypass with autologous tissue substitute, open approach
0D1B0JP	Bypass of ileum to rectum with synthetic substitute, open approach
0D1B0KP	Ileum to rectum bypass with non-autologous tissue substitute, open approach
0D1B0ZP	Bypass from ileum to rectum, open approach
0D1B47P	Ileum to rectum bypass with autologous tissue substitute, percutaneous endoscopic approach
0D1B4JP	Ileum to rectum bypass with synthetic substitute, percutaneous endoscopic approach
0D1B4KP	Ileum to rectum bypass with non-autologous tissue substitute, percutaneous endoscopic approach
0D1B4ZP	Ileum to rectum bypass, endoscopic percutaneous approach
0D1B87P	Ileum to rectum bypass with autologous tissue substitute, endoscopic natural or artificial orifice approach
0D1B8JP	Ileum to rectum bypass with synthetic substitute, natural or artificial orifice endoscopic approach
0D1B8KP	Ileum to rectum bypass with non-autologous tissue substitute, endoscopic natural or artificial orifice approach
0D1B8ZP	Ileum to rectum bypass, natural orifice or endoscopic artificial orifice approach
0D1H87P	Cecum to rectum bypass with autologous tissue substitute, natural orifice or endoscopic artificial orifice approach
0D1H8JP	Cecum to rectum bypass with synthetic substitute, natural orifice or endoscopic artificial orifice approach
0D1H8KP	Cecum to rectum bypass with non-autologous tissue substitute, natural orifice or endoscopic artificial orifice approach
0D1H8ZP	Cecum to rectum shunt, natural or artificial orifice endoscopic approach

0D1B07H	Ileum to cecum bypass with autologous tissue substitute, open approach
0D1B07K	Ileum to ascending colon bypass with autologous tissue substitute, open approach
0D1B07L	Ileum to transverse colon bypass with autologous tissue substitute, open approach
0D1B07M	Ileum to descending colon bypass with autologous tissue substitute, open approach
0D1B07N	Ileum to sigmoid colon bypass with autologous tissue substitute, open approach
0D1B0JH	Ileum to cecum bypass with synthetic substitute, open approach
0D1B0JK	Bypass of ileum to ascending colon with synthetic substitute, open approach
0D1B0JL	Bypass of ileum to transverse colon with synthetic substitute, open approach
0D1B0JM	Bypass of ileum to descending colon with synthetic substitute, open approach
0D1B0JN	Bypass of ileum to sigmoid colon with synthetic substitute, open approach
0D1B0KH	Ileum to cecum bypass with non-autologous tissue substitute, open approach
0D1B0KK	Ileum to ascending colon bypass with non-autologous tissue substitute, open approach
0D1B0KL	Ileum to transverse colon bypass with non-autologous tissue substitute, open approach
0D1B0KM	Ileum to descending colon bypass with non-autologous tissue substitute, open approach
0D1B0KN	Ileum to sigmoid colon bypass with non-autologous tissue substitute, open approach
0D1B0ZH	Ileum to cecum bypass, open approach
0D1B0ZK	Bypass from ileum to ascending colon, open approach
0D1B0ZL	Ileum to transverse colon shunt, open approach
0D1B0ZM	Bypass from ileum to descending colon, open approach
0D1B0ZN	Bypass from ileum to sigmoid colon, open approach
0D1B47H	Ileum to cecum bypass with autologous tissue substitute, percutaneous endoscopic approach
0D1B47K	Bypass of ileum to ascending colon with autologous tissue substitute, percutaneous endoscopic approach
0D1B47L	Bypass of ileum to transverse colon with autologous tissue substitute, percutaneous endoscopic approach
0D1B47M	Bypass of ileum to descending colon with autologous tissue substitute, percutaneous endoscopic approach
0D1B47N	Bypass of ileum to sigmoid colon with autologous tissue substitute, percutaneous endoscopic approach
0D1B4JH	Ileum to cecum bypass with synthetic substitute, percutaneous endoscopic approach
0D1B4JK	Bypass of ileum to ascending colon with synthetic substitute, percutaneous endoscopic approach
0D1B4JL	Bypass of ileum to transverse colon with synthetic substitute, percutaneous endoscopic approach
0D1B4JM	Bypass of ileum to descending colon with synthetic substitute, percutaneous endoscopic approach
0D1B4JN	Bypass of ileum to sigmoid colon with synthetic substitute, percutaneous endoscopic approach
0D1B4KH	Ileum to cecum bypass with non-autologous tissue substitute, percutaneous endoscopic approach
0D1B4KK	Bypass of ileum to ascending colon with non-autologous tissue substitute, percutaneous endoscopic approach
0D1B4KL	Bypass of ileum to transverse colon with non-autologous tissue substitute, percutaneous endoscopic approach

0D1B4KM	Bypass of ileum to descending colon with non-autologous tissue substitute, percutaneous endoscopic approach
0D1B4KN	Bypass of ileum to sigmoid colon with non-autologous tissue substitute, percutaneous endoscopic approach
0D1B4ZH	Ileum to cecum bypass, percutaneous endoscopic approach
0D1B4ZK	Bypass from ileum to ascending colon, percutaneous endoscopic approach
0D1B4ZL	Bypass from ileum to transverse colon, percutaneous endoscopic approach
0D1B4ZM	Bypass from ileum to descending colon, percutaneous endoscopic approach
0D1B4ZN	Bypass of ileum to sigmoid colon, percutaneous endoscopic approach
0D1B87H	Ileum to cecum bypass with autologous tissue substitute, natural orifice or endoscopic artificial orifice approach
0D1B87K	Ileum to ascending colon bypass with autologous tissue substitute, natural orifice or endoscopic artificial orifice approach
0D1B87L	Ileum to transverse colon bypass with autologous tissue substitute, natural or artificial orifice endoscopic approach
0D1B87M	Ileum to descending colon bypass with autologous tissue substitute, natural orifice or endoscopic artificial orifice approach
0D1B87N	Ileum to sigmoid colon bypass with autologous tissue substitute, natural or artificial endoscopic orifice approach
0D1B8JH	Ileum to cecum bypass with synthetic substitute, natural orifice or endoscopic artificial orifice approach
0D1B8JK	Bypass of ileum to ascending colon with synthetic substitute, natural orifice or endoscopic artificial orifice approach
0D1B8JL	Bypass of ileum to transverse colon with synthetic substitute, natural orifice or endoscopic artificial orifice approach
0D1B8JM	Bypass of ileum to descending colon with synthetic substitute, natural orifice or endoscopic artificial orifice approach
0D1B8JN	Bypass of ileum to sigmoid colon with synthetic substitute, natural orifice or endoscopic artificial orifice approach
0D1B8KH	Ileum to cecum bypass with non-autologous tissue substitute, natural orifice or endoscopic artificial orifice approach
0D1B8KK	Ileum to ascending colon bypass with non-autologous tissue substitute, endoscopic natural or artificial orifice approach
0D1B8KL	Ileum to transverse colon bypass with non-autologous tissue substitute, natural or artificial endoscopic orifice approach
0D1B8KM	Ileum to descending colon bypass with non-autologous tissue substitute, natural orifice or endoscopic artificial orifice approach
0D1B8KN	Ileum to sigmoid colon bypass with non-autologous tissue substitute, endoscopic natural or artificial orifice approach
0D1B8ZK	Ileum to ascending colon bypass, natural orifice or endoscopic artificial orifice approach
0D1B8ZL	Bypass from ileum to transverse colon, natural or artificial endoscopic orifice approach
0D1B8ZM	Bypass from ileum to descending colon, natural orifice or endoscopic artificial orifice approach
0D1B8ZN	Bypass from ileum to sigmoid colon, natural or artificial endoscopic orifice approach
0D1H07H	Cecum to cecum bypass with autologous tissue substitute, open approach
0D1H07K	Cecum to ascending colon bypass with autologous tissue substitute, open approach
0D1H07L	Cecum to transverse colon bypass with autologous tissue substitute, open approach
0D1H07M	Cecum to descending colon bypass with autologous tissue substitute, open approach
0D1H07N	Cecum to sigmoid colon bypass with autologous tissue substitute, open approach

0D1H07P	Cecum to rectum bypass with autologous tissue substitute, open approach
0D1H0JH	Cecum to cecum bypass with synthetic substitute, open approach
0D1H0JK	Cecum to ascending colon bypass with synthetic substitute, open approach
0D1H0JL	Cecum to transverse colon bypass with synthetic substitute, open approach
0D1H0JM	Cecum to descending colon bypass with synthetic substitute, open approach
0D1H0JN	Cecum to sigmoid colon bypass with synthetic substitute, open approach
0D1H0JP	Cecum to rectum bypass with synthetic substitute, open approach
0D1H0KH	Cecum to cecum bypass with non-autologous tissue substitute, open approach
0D1H0KK	Cecum to ascending colon bypass with non-autologous tissue substitute, open approach
0D1H0KL	Cecum to transverse colon bypass with non-autologous tissue substitute, open approach
0D1H0KM	Cecum to descending colon bypass with non-autologous tissue substitute, open approach
0D1H0KN	Cecum to sigmoid colon bypass with non-autologous tissue substitute, open approach
0D1H0KP	Cecum to rectum bypass with non-autologous tissue substitute, open approach
0D1H0ZH	Cecum to cecum shunt, open approach
0D1H0ZK	Cecum to ascending colon shunt, open approach
0D1H0ZL	Cecum to transverse colon shunt, open approach
0D1H0ZM	Cecum to descending colon shunt, open approach
0D1H0ZN	Cecum to sigmoid colon bypass, open approach
0D1H0ZP	Cecum to rectum shunt, open approach
0D1H47H	Cecum to cecum bypass with autologous tissue substitute, percutaneous endoscopic approach
0D1H47K	Cecum to ascending colon bypass with autologous tissue substitute, percutaneous endoscopic approach
0D1H47L	Cecum to transverse colon bypass with autologous tissue substitute, percutaneous endoscopic approach
0D1H47M	Cecum to descending colon bypass with autologous tissue substitute, percutaneous endoscopic approach
0D1H47N	Cecum to sigmoid colon bypass with autologous tissue substitute, percutaneous endoscopic approach
0D1H47P	Cecum to rectum bypass with autologous tissue substitute, percutaneous endoscopic approach
0D1H4JH	Cecum to cecum bypass with synthetic substitute, percutaneous endoscopic approach
0D1H4JK	Cecum to ascending colon bypass with synthetic substitute, percutaneous endoscopic approach
0D1H4JL	Cecum to transverse colon bypass with synthetic substitute, percutaneous endoscopic approach
0D1H4JM	Cecum to descending colon bypass with synthetic substitute, percutaneous endoscopic approach
0D1H4JN	Cecum to sigmoid colon bypass with synthetic substitute, percutaneous endoscopic approach
0D1H4JP	Cecum to rectum bypass with synthetic substitute, percutaneous endoscopic approach
0D1H4KH	Cecum to cecum bypass with non-autologous tissue substitute, percutaneous endoscopic approach
0D1H4KK	Cecum to ascending colon bypass with non-autologous tissue substitute, percutaneous endoscopic approach

0D1H4KL	Cecum to transverse colon bypass with non-autologous tissue substitute, percutaneous endoscopic approach
0D1H4KM	Cecum to descending colon bypass with non-autologous tissue substitute, percutaneous endoscopic approach
0D1H4KN	Cecum to sigmoid colon bypass with non-autologous tissue substitute, percutaneous endoscopic approach
0D1H4KP	Cecum to rectum bypass with non-autologous tissue substitute, percutaneous endoscopic approach
0D1H4ZH	Cecum to cecum bypass, endoscopic percutaneous approach
0D1H4ZK	Cecum to ascending colon bypass, endoscopic percutaneous approach
0D1H4ZL	Cecum to transverse colon bypass, endoscopic percutaneous approach
0D1H4ZM	Cecum to descending colon bypass, endoscopic percutaneous approach
0D1H4ZN	Cecum to sigmoid colon bypass, percutaneous endoscopic approach
0D1H4ZP	Cecum to rectum bypass, endoscopic percutaneous approach
0D1H87H	Cecum to cecum bypass with autologous tissue substitute, natural orifice or endoscopic artificial orifice approach
0D1H87K	Cecum to ascending colon bypass with autologous tissue substitute, natural orifice or endoscopic artificial orifice approach
0D1H87L	Cecum to transverse colon bypass with autologous tissue substitute, natural orifice or endoscopic artificial orifice approach
0D1H87M	Cecum to descending colon bypass with autologous tissue substitute, natural orifice or endoscopic artificial orifice approach
0D1H87N	Cecum to sigmoid colon bypass with autologous tissue substitute, natural orifice or endoscopic artificial orifice approach
0D1H8JH	Cecum to cecum bypass with synthetic substitute, natural orifice or endoscopic artificial orifice approach
0D1H8JK	Cecum to ascending colon bypass with synthetic substitute, natural orifice or endoscopic artificial orifice approach
0D1H8JL	Cecum to transverse colon bypass with synthetic substitute, natural orifice or endoscopic artificial orifice approach
0D1H8JM	Cecum to descending colon bypass with synthetic substitute, natural orifice or endoscopic artificial orifice approach
0D1H8JN	Cecum to sigmoid colon bypass with synthetic substitute, natural orifice or endoscopic artificial orifice approach
0D1H8KH	Cecum to cecum bypass with non-autologous tissue substitute, natural orifice or endoscopic artificial orifice approach
0D1H8KK	Cecum to ascending colon bypass with non-autologous tissue substitute, natural orifice or endoscopic artificial orifice approach
0D1H8KL	Cecum to transverse colon bypass with non-autologous tissue substitute, natural or artificial endoscopic orifice approach
0D1H8KM	Cecum to descending colon bypass with non-autologous tissue substitute, natural orifice or endoscopic artificial orifice approach
0D1H8KN	Cecum to sigmoid colon bypass with non-autologous tissue substitute, natural or artificial endoscopic orifice approach
0D1H8ZH	Cecum to cecum shunt, natural or artificial orifice endoscopic approach
0D1H8ZK	Cecum to ascending colon bypass, natural orifice or endoscopic artificial orifice approach
0D1H8ZL	Cecum to transverse colon bypass, natural orifice or endoscopic artificial orifice approach
0D1H8ZM	Cecum to descending colon bypass, natural orifice or endoscopic artificial orifice approach
0D1H8ZN	Cecum to sigmoid colon bypass, natural orifice or endoscopic artificial orifice approach
0D1K07K	Ascending colon to ascending colon bypass with autologous tissue substitute, open approach

0D1K07L	Bypass of ascending colon to transverse colon with autologous tissue substitute, open approach
0D1K07M	Bypass from ascending to descending colon with autologous tissue substitute, open approach
0D1K07N	Bypass of ascending colon to sigmoid colon with autologous tissue substitute, open Approach
0D1K07P	Bypass of ascending colon to rectum with autologous tissue substitute, open approach
0D1K0JK	Bypass from ascending colon to ascending colon with synthetic substitute, open approach
0D1K0JL	Bypass of ascending colon to transverse colon with synthetic substitute, open approach
0D1K0JM	Bypass from ascending to descending colon with synthetic substitute, open approach
0D1K0JN	Bypass of ascending colon to sigmoid colon with synthetic substitute, open approach
0D1K0JP	Bypass of ascending colon to rectum with synthetic substitute, open approach
0D1K0KK	Ascending colon to ascending colon bypass with non-autologous tissue substitute, open approach
0D1K0KL	Bypass of ascending colon to transverse colon with non-autologous tissue substitute, open approach
0D1K0KM	Bypass from ascending to descending colon with non-autologous tissue substitute, open approach
0D1K0KN	Bypass of ascending colon to sigmoid colon with non-autologous tissue substitute, open approach
0D1K0KP	Ascending colon to rectum bypass with non-autologous tissue substitute, open approach
0D1K0ZK	Ascending colon to ascending colon bypass, open approach
0D1K0ZL	Bypass of ascending colon to transverse colon, open approach
0D1K0ZM	Ascending to descending colon bypass, open approach
0D1K0ZN	Bypass from ascending to sigmoid colon, open approach
0D1K0ZP	Ascending colon to rectum bypass, open approach
0D1K47K	Bypass of ascending colon to ascending colon with autologous tissue substitute, percutaneous endoscopic approach
0D1K47L	Bypass of ascending colon to transverse colon with autologous tissue substitute, percutaneous endoscopic approach
0D1K47M	Bypass from ascending to descending colon with autologous tissue substitute, percutaneous endoscopic approach
0D1K47N	Bypass of ascending colon to sigmoid colon with autologous tissue substitute, percutaneous endoscopic approach
0D1K47P	Bypass of ascending colon to rectum with autologous tissue substitute, percutaneous endoscopic approach
0D1K4JK	Bypass of ascending colon to ascending colon with synthetic substitute, percutaneous endoscopic approach
0D1K4JL	Bypass of ascending colon to transverse colon with synthetic substitute, percutaneous endoscopic approach
0D1K4JM	Bypass from ascending to descending colon with synthetic substitute, percutaneous endoscopic approach
0D1K4JN	Bypass of ascending colon to sigmoid colon with synthetic substitute, percutaneous endoscopic approach
0D1K4JP	Bypass of ascending colon to rectum with synthetic substitute, percutaneous endoscopic approach
0D1K4KK	Ascending colon to ascending colon bypass with non-autologous tissue substitute, percutaneous endoscopic approach
0D1K4KL	Bypass of ascending colon to transverse colon with non-autologous tissue substitute, percutaneous endoscopic approach

0D1K4KM	Bypass from ascending to descending colon with non-autologous tissue substitute, percutaneous endoscopic approach
0D1K4KN	Bypass of ascending colon to sigmoid colon with non-autologous tissue substitute, percutaneous endoscopic approach
0D1K4KP	Bypass of ascending colon to rectum with non-autologous tissue substitute, percutaneous endoscopic approach
0D1K4ZK	Bypass from ascending colon to ascending colon, percutaneous endoscopic approach
0D1K4ZL	Bypass of ascending colon to transverse colon, percutaneous endoscopic approach
0D1K4ZM	Bypass from ascending to descending colon, percutaneous endoscopic approach
0D1K4ZN	Bypass of ascending colon to sigmoid colon, percutaneous endoscopic approach
0D1K4ZP	Bypass of ascending colon to rectum, percutaneous endoscopic approach
0D1K87K	Ascending colon to ascending colon bypass with autologous tissue substitute, natural orifice or endoscopic artificial orifice approach
0D1K87L	Ascending to transverse colon bypass with autologous tissue substitute, natural orifice or endoscopic artificial orifice approach
0D1K87M	Ascending to descending colon bypass with autologous tissue substitute, natural orifice or endoscopic artificial orifice approach
0D1K87N	Bypass of ascending colon to sigmoid colon with autologous tissue substitute, endoscopic natural or artificial orifice approach
0D1K87P	Ascending colon to rectum bypass with autologous tissue substitute, natural or artificial endoscopic orifice approach
0D1K8JK	Ascending colon to ascending colon bypass with synthetic substitute, natural orifice or endoscopic artificial orifice approach
0D1K8JL	Bypass of ascending colon to transverse colon with synthetic substitute, natural orifice or endoscopic artificial orifice approach
0D1K8JM	Bypass from ascending to descending colon with synthetic substitute, natural orifice or endoscopic artificial orifice approach
0D1K8JN	Bypass of ascending colon to sigmoid colon with synthetic substitute, natural orifice or endoscopic artificial orifice approach
0D1K8JP	Bypass of ascending colon to rectum with synthetic substitute, natural or artificial endoscopic orifice approach
0D1K8KK	Ascending colon to ascending colon bypass with non-autologous tissue substitute, natural orifice or endoscopic artificial orifice approach
0D1K8KL	Ascending to transverse colon bypass with non-autologous tissue substitute, natural or artificial endoscopic orifice approach
0D1K8KM	Ascending to descending colon bypass with non-autologous tissue substitute, natural orifice or endoscopic artificial orifice approach
0D1K8KN	Bypass of ascending colon to sigmoid colon with non-autologous tissue substitute, endoscopic natural or artificial orifice approach
0D1K8KP	Ascending colon to rectum bypass with non-autologous tissue substitute, natural orifice or endoscopic artificial orifice approach
0D1K8ZK	Ascending colon to ascending colon bypass, natural orifice or endoscopic artificial orifice approach
0D1K8ZL	Bypass of ascending colon to transverse colon, natural orifice or endoscopic artificial orifice approach
0D1K8ZM	Bypass from ascending to descending colon, natural orifice or endoscopic artificial orifice approach
0D1K8ZN	Bypass of ascending colon to sigmoid colon, natural or artificial endoscopic orifice approach
0D1K8ZP	Ascending colon to rectum bypass, natural orifice or endoscopic artificial orifice approach
0D1L07L	Transverse to transverse colon bypass with autologous tissue substitute, open approach
0D1L07M	Transverse to descending colon bypass with autologous tissue substitute, open approach

0D1L07N	Transverse to sigmoid colon bypass with autologous tissue substitute, open approach
0D1L07P	Transverse colon to rectal shunt with autologous tissue substitute, open approach
0D1L0JL	Transverse colon to transverse colon bypass with synthetic substitute, open approach
0D1L0JM	Transverse to descending colon bypass with synthetic substitute, open approach
0D1L0JN	Transverse to sigmoid colon bypass with synthetic substitute, open approach
0D1L0JP	Transverse colon to rectal shunt with synthetic substitute, open approach
0D1L0KL	Transverse to transverse colon bypass with non-autologous tissue substitute, open Approach
0D1L0KM	Transverse to descending colon bypass with non-autologous tissue substitute, open approach
0D1L0KN	Transverse to sigmoid colon bypass with non-autologous tissue substitute, open approach
0D1L0ZL	Transverse to transverse colon shunt, open approach
0D1L0ZM	Transverse to descending colon shunt, open approach
0D1L0ZN	Transverse to sigmoid colon bypass, open approach
0D1L0ZP	Transverse colon to rectum shunt, open approach
0D1L47L	Transverse to transverse colon bypass with autologous tissue substitute, percutaneous endoscopic approach
0D1L47M	Transverse to descending colon bypass with autologous tissue substitute, percutaneous endoscopic approach
0D1L47N	Transverse colon to sigmoid colon bypass with autologous tissue substitute, percutaneous endoscopic approach
0D1L4JL	Transverse to transverse colon bypass with synthetic substitute, percutaneous endoscopic approach
0D1L4JM	Transverse to descending colon bypass with synthetic substitute, percutaneous endoscopic approach
0D1L4JN	Transverse to sigmoid colon bypass with synthetic substitute, percutaneous endoscopic approach
0D1L4JP	Transverse colon to rectum bypass with synthetic substitute, percutaneous endoscopic approach
0D1L4KL	Transverse colon to transverse colon bypass with non-autologous tissue substitute, percutaneous endoscopic approach
0D1L4KM	Transverse to descending colon bypass with non-autologous tissue substitute, percutaneous endoscopic approach
0D1L4KN	Transverse colon to sigmoid colon bypass with non-autologous tissue substitute, percutaneous endoscopic approach
0D1L4KP	Transverse colon to rectal bypass with non-autologous tissue substitute, percutaneous endoscopic approach
0D1L4ZL	Transverse to transverse colon bypass, percutaneous endoscopic approach
0D1L4ZM	Transverse to descending colon bypass, percutaneous endoscopic approach
0D1L4ZN	Transverse to sigmoid colon bypass, percutaneous endoscopic approach
0D1L4ZP	Transverse colon to rectum bypass, percutaneous endoscopic approach
0D1L87L	Transverse to transverse colon bypass with autologous tissue substitute, natural or artificial orifice endoscopic approach
0D1L87M	Transverse to descending colon bypass with autologous tissue substitute, natural orifice or endoscopic artificial orifice approach
0D1L87N	Transverse to sigmoid colon bypass with autologous tissue substitute, natural orifice or endoscopic artificial orifice approach
0D1L87P	Transverse colon to rectal bypass with autologous tissue substitute, natural orifice or endoscopic artificial orifice approach

0D1L8JL	Transverse to transverse colon bypass with synthetic substitute, natural orifice or endoscopic artificial orifice approach
0D1L8JM	Transverse to descending colon bypass with synthetic substitute, natural orifice or endoscopic artificial orifice approach
0D1L8JN	Transverse to sigmoid colon bypass with synthetic substitute, natural orifice or endoscopic artificial orifice approach
0D1L8JP	Transverse colon to rectal bypass with synthetic substitute, natural or artificial orifice endoscopic approach
0D1L8KL	Transverse to transverse colon bypass with non-autologous tissue substitute, natural orifice or endoscopic artificial orifice approach
0D1L8KM	Transverse to descending colon bypass with non-autologous tissue substitute, natural or artificial orifice endoscopic approach
0D1L8KN	Transverse to sigmoid colon bypass with non-autologous tissue substitute, natural or artificial orifice endoscopic approach
0D1L8KP	Transverse colon to rectal bypass with non-autologous tissue substitute, endoscopic natural or artificial orifice approach
0D1L8ZL	Transverse to transverse colon bypass, endoscopic natural or artificial orifice approach
0D1L8ZM	Transverse to descending colon shunt, natural or artificial endoscopic orifice approach
0D1L8ZN	Transverse colon to sigmoid colon bypass, natural or artificial endoscopic orifice approach
0D1L8ZP	Transverse colon to rectum bypass, natural or artificial orifice endoscopic approach
0D1M07M	Descending colon to descending colon bypass with autologous tissue substitute, open approach
0D1M07N	Descending to sigmoid colon bypass with autologous tissue substitute, open approach
0D1M07P	Descending colon to rectum bypass with autologous tissue substitute, open approach
0D1M0JM	Descending colon to descending colon bypass with synthetic substitute, open approach
0D1M0JN	Bypass of descending colon to sigmoid colon with synthetic substitute, open approach
0D1M0JP	Descending colon to rectal shunt with synthetic substitute, open approach
0D1M0KM	Descending colon to descending colon bypass with non-autologous tissue substitute, open approach
0D1M0KN	Descending to sigmoid colon bypass with non-autologous tissue substitute, open approach
0D1M0KP	Descending colon to rectum bypass with non-autologous tissue substitute, open approach
0D1M0ZM	Descending colon to descending colon bypass, open approach
0D1M0ZN	Descending to sigmoid colon shunt, open approach
0D1M0ZP	Descending colon to rectum bypass, open approach
0D1M47M	Descending colon to descending colon bypass with autologous tissue substitute, percutaneous endoscopic approach
0D1M47N	Bypass of descending colon to sigmoid colon with autologous tissue substitute, percutaneous endoscopic approach
0D1M47P	Descending colon to rectum bypass with autologous tissue substitute, percutaneous endoscopic approach
0D1M4JM	Descending colon to descending colon bypass with synthetic substitute, percutaneous endoscopic approach
0D1M4JN	Bypass of descending colon to sigmoid colon with synthetic substitute, percutaneous endoscopic approach
0D1M4JP	Bypass of descending colon to rectum with synthetic substitute, percutaneous endoscopic approach
0D1M4KM	Descending colon to descending colon bypass with non-autologous tissue substitute, percutaneous endoscopic approach

0D1M4KN	Bypass of descending colon to sigmoid colon with non-autologous tissue substitute, percutaneous endoscopic approach
0D1M4KP	Descending colon to rectum bypass with non-autologous tissue substitute, percutaneous endoscopic approach
0D1M4ZM	Descending colon to descending colon bypass, endoscopic percutaneous approach
0D1M4ZN	Bypass of descending colon to sigmoid colon, percutaneous endoscopic approach
0D1M4ZP	Descending colon to rectum bypass, percutaneous endoscopic approach
0D1M87M	Descending colon to descending colon bypass with autologous tissue substitute, natural orifice or endoscopic artificial orifice approach
0D1M87N	Descending to sigmoid colon bypass with autologous tissue substitute, natural orifice or endoscopic artificial orifice approach
0D1M87P	Descending colon to rectum bypass with autologous tissue substitute, endoscopic natural or artificial orifice approach
0D1M8JM	Descending colon to descending colon bypass with synthetic substitute, natural orifice or endoscopic artificial orifice approach
0D1M8JN	Bypass of descending colon to sigmoid colon with synthetic substitute, natural orifice or endoscopic artificial orifice approach
0D1M8JP	Bypass of descending colon to rectum with synthetic substitute tic, natural or artificial endoscopic orifice approach
0D1M8KM	Descending colon to descending colon bypass with non-autologous tissue substitute, natural orifice or endoscopic artificial orifice approach
0D1M8KN	Descending colon to sigmoid colon bypass with non-autologous tissue substitute, natural or artificial endoscopic orifice approach
0D1M8KP	Descending colon to rectal bypass with non-autologous tissue substitute, natural orifice or endoscopic artificial orifice approach
0D1M8ZM	Descending colon to descending colon bypass, natural orifice or endoscopic artificial orifice approach
0D1M8ZN	Bypass of descending colon to sigmoid colon, natural or artificial endoscopic orifice approach
0D1M8ZP	Descending colon to rectum bypass, natural or artificial orifice endoscopic approach
0D1N07N	Sigmoid to sigmoid colon bypass with autologous tissue substitute, open approach
0D1N07P	Sigmoid colon to rectal bypass with autologous tissue substitute, open approach
0D1N0JN	Sigmoid to sigmoid colon bypass with synthetic substitute, open approach
0D1N0JP	Sigmoid colon to rectal bypass with synthetic substitute, open approach
0D1N0KN	Sigmoid to sigmoid colon bypass with non-autologous tissue substitute, open approach
0D1N0KP	Sigmoid colon to rectal bypass with non-autologous tissue substitute, open approach
0D1N0ZN	Sigmoid to sigmoid colon shunt, open approach
0D1N0ZP	Sigmoid colon to rectum bypass, open approach
0D1N47N	Sigmoid to sigmoid colon bypass with autologous tissue substitute, percutaneous endoscopic approach
0D1N47P	Sigmoid colon to rectum bypass with autologous tissue substitute, percutaneous endoscopic approach
0D1N4JN	Sigmoid to sigmoid colon bypass with synthetic substitute, percutaneous endoscopic approach
0D1N4JP	Sigmoid colon to rectum bypass with synthetic substitute, percutaneous endoscopic approach
0D1N4KN	Sigmoid to sigmoid colon bypass with non-autologous tissue substitute, percutaneous endoscopic approach
0D1N4KP	Sigmoid colon to rectum bypass with non-autologous tissue substitute, percutaneous endoscopic approach

0D1N4ZN	Sigmoid to sigmoid colon bypass, percutaneous endoscopic approach
0D1N4ZP	Sigmoid colon to rectum bypass, percutaneous endoscopic approach
0D1N87N	Sigmoid to sigmoid colon bypass with autologous tissue substitute, natural or artificial orifice endoscopic approach
0D1N87P	Sigmoid colon to rectal bypass with autologous tissue substitute, natural orifice or endoscopic artificial orifice approach
0D1N8JN	Sigmoid to sigmoid colon bypass with synthetic substitute, natural orifice or endoscopic artificial orifice approach
0D1N8JP	Sigmoid colon to rectum bypass with synthetic substitute, natural or artificial endoscopic orifice approach
0D1N8KN	Sigmoid to sigmoid colon bypass with non-autologous tissue substitute, natural or artificial endoscopic orifice approach
0D1N8KP	Sigmoid colon to rectal bypass with non-autologous tissue substitute, natural orifice or endoscopic artificial orifice approach
0D1N8ZN	Sigmoid to sigmoid colon bypass, natural or artificial orifice endoscopic approach
0D1N8ZP	Sigmoid colon to rectum bypass, natural orifice or endoscopic artificial orifice approach
0D1B07Q	Ileum to anus bypass with autologous tissue substitute, open approach
0D1B0JQ	Bypass of ileum to anus with synthetic substitute, open approach
0D1B0KQ	Ileum to anus bypass with non-autologous tissue substitute, open approach
0D1B0ZQ	Ileum to anus shunt, open approach
0D1B47Q	Ileum to anus bypass with autologous tissue substitute, percutaneous endoscopic approach
0D1B4JQ	Bypass of ileum to anus with synthetic substitute, percutaneous endoscopic approach
0D1B4KQ	Ileum to anus bypass with non-autologous tissue substitute, percutaneous endoscopic approach
0D1B4ZQ	Ileum to anus shunt, percutaneous endoscopic approach
0D1B87Q	Ileum to anus bypass with autologous tissue substitute, endoscopic natural or artificial orifice approach
0D1B8JQ	Ileum to anus bypass with synthetic substitute, natural or artificial orifice endoscopic approach
0D1B8KQ	Ileum to anus bypass with non-autologous tissue substitute, natural orifice or endoscopic artificial orifice approach
0D1B8ZQ	Ileum to anus bypass, natural orifice or endoscopic artificial orifice approach
0DBJ0ZX	Excision in appendix, open approach, diagnostic
0DBJ0ZZ	Appendix excision, open approach
0D9Q00Z	Drainage in anus with drainage device, open approach
0D9Q0ZZ	Drainage in anus, open approach
0D9Q30Z	Drainage in anus with drainage device, percutaneous approach
0D9Q3ZZ	Drainage in anus, percutaneous approach
0D9Q40Z	Anal drainage with drainage device, endoscopic percutaneous approach
0D9Q4ZZ	Anal drainage, endoscopic percutaneous approach
0D9Q70Z	Drainage into anus with drainage device, natural or artificial orifice approach
0D9Q7ZZ	Drainage in the anus, natural or artificial orifice approach
0D9Q80Z	Drainage in anus with drainage device, natural or artificial orifice endoscopic approach
0D5Q0ZZ	Destruction in anus, open approach
0D5Q3ZZ	Destruction in anus, percutaneous approach
0D5Q7ZZ	Destruction in anus, natural or artificial orifice approach

0D5QXZZ	Destruction in anus, external approach
0D5R0ZZ	Destruction in anal sphincter, open approach
0D5R3ZZ	Anal sphincter destruction, percutaneous approach

Supplementary Table S4. ICD-10 codes for hospitalizations.

CODE	DESCRIPTION
A00–B99	Infectious and parasitic diseases
C00–D48	Neoplasms
D50–D89	Diseases of the blood and hematopoietic organs
E00–E90	Endocrine, nutritional and metabolic diseases
F00–F99	Mental and behavioral disorders
G00–G99	Diseases of the nervous system
I00–I99	Diseases of the circulatory system
J00–J99	Diseases of the respiratory system
K00–K93	Diseases of the digestive system
K50-K51	Inflammatory bowel disease
N00–N99	Diseases of the genitourinary system
S00–T98	Injuries, poisoning and other external causes
R00–R99	Symptoms, signs and abnormal clinical findings not elsewhere classified

Supplementary Table S5. Use of drugs in IBD from 2017 to 2023. Absolute number and percentage of IBD patients treated.

	Population of Catalonia	IBD patients	Immunosuppressant treatment		Salicylates		Biological treatment		Corticoids	
			**Number**	**%**	**Number**	**%**	**Number**	**%**	**Number**	**%**
2017	7,496,276	28,754	6,282	21.8	10,466	36.4	3,873	13.5	2,542	8.8
2018	7,543,825	32,174	6,712	20.9	11,506	35.8	4,579	14.2	2,820	8.8
2019	7,619,494	34,567	6,993	20.2	12,566	36.4	5,335	15.4	3,198	9.3
2020	7,722,203	36,182	7,046	19.5	13,313	36.8	5,955	16.5	2,920	8.1
2021	7,739,758	38,059	7,302	19.2	13,945	36.6	6,787	17.8	3,368	8.8
2022	7,758,615	39,778	7,295	18.3	14,487	36.4	7,697	19.3	3,650	9.2
2023	8,016,606	41,423	7,223	17.4	15,136	36.5	8,709	21.0	3,966	9.6

Supplementary Table S6. Use of drugs in CD from 2017 to 2023. Absolute number and percentage of CD patients treated.

	Population of Catalonia	CD patients	Immunosuppressant treatment		Salicylates		Biological treatment		Corticoids	
			Number	%	Number	%	Number	%	Number	%
2017	7,496,276	11,219	4,128	36.8	1,805	16.1	2,814	25.1	1,036	9.2
2018	7,543,825	12,426	4,347	35.0	1,879	15.1	3,335	26.8	1,127	9.1
2019	7,619,494	13,304	4,466	33.6	2,007	15.1	3,838	28.8	1,297	9.7
2020	7,722,203	13,880	4,445	32.0	2,004	14.4	4,255	30.7	1,177	8.5
2021	7,739,758	14,527	4,577	31.5	2,017	13.9	4,786	32.9	1,325	9.1
2022	7,758,615	15,133	4,525	29.9	2,077	13.7	5,379	35.5	1,436	9.5
2023	8,016,606	15,694	4,391	28.0	2,065	13.2	5,989	38.2	1,552	9.9

Supplementary Table S7. Use of drugs in UC from 2017 to 2023. Absolute number and percentage of UC patients treated.

	Population of Catalonia	UC Patients	Immunosuppressant treatment		Salicylates		Biological treatment		Corticoids	
			Number	%	Number	%	Number	%	Number	%
2017	7,496,276	17,534	2,154	12.3	8,661	49.4	1,059	6	1,506	8.6
2018	7,543,825	19,747	2,365	12.0	9,627	48.8	1,244	6.3	1,693	8.6
2019	7,619,494	21,262	2,527	11.9	10,559	49.7	1,497	7	1,901	8.9
2020	7,722,203	22,301	2,601	11.7	11,309	50.7	1,700	7.6	1,743	7.8
2021	7,739,758	23,531	2,725	11.6	11,928	50.7	2,001	8.5	2,043	8.7
2022	7,758,615	24,644	2,770	11.2	12,410	50.4	2,318	9.4	2,214	9
2023	8,016,606	25,728	2,832	11.0	13,071	50.8	2,720	10.6	2,414	9.4

Supplementary Table S8. Biologic treatment in IBD patients. Percentages are calculated by dividing the number of patients receiving the drug by the total number of patients with IBD

	**Population of Catalonia**	**IBD patients**	IFX		ADA		USTE		GOLI		VEDO		UPA		TOFA		FILGO	
			**Number**	**%**	**Number**	**%**	Number	**%**	**Number**	**%**	**Number**	**%**	**Number**	**%**	**Number**	**%**	**Number**	**%**
2017	7,496,276	28,754	1,807	6.3	1,795	6.2	133	0.5	135	0.5	275	1	0	0	2	0.01	0	0
2018	7,543,825	32,174	1,930	6	2,082	6.5	400	1.2	165	0.5	393	1.2	0	0	22	1	0	0
2019	7,619,494	34,567	2,051	5.9	2,333	6.7	708	2.0	176	0.5	504	1.5	0	0	62	2	0	0
2020	7,722,203	36,182	2,088	5.8	2,523	7	955	2.6	155	0.4	613	1.7	0	0	120	3	0	0
2021	7,739,758	38,059	2,280	6	2,794	7.3	1,258	3.3	161	0.4	699	1.8	7	0	150	4	0	0
2022	7,758,615	39,778	2,549	6.4	3,034	7.6	1,668	4.2	162	0.4	751	1.9	29	0.1	159	4	0	0
2023	8,016,606	41,423	2,865	6.9	3,281	7.9	2,001	4.8	161	0.4	831	2	104	0.3	185	4	19	0.1

Supplementary Table S9. Biologic treatment in CD patients. Percentages are calculated by dividing the number of patients receiving the drug by the total number of patients with CD

	Population of Catalonia	CD patients	IFX		ADA		USTE		GOLI		VEDO		UPA		TOFA		FILGO	
			**Number**	**%**	**Number**	**%**	**Number**	**%**	**Number**	**%**	**Number**	**%**	**Number**	**%**	**Number**	**%**	**Number**	**%**
2017	7,496,276	11,219	1210	10.8	1,499	13.4	116	1	24	0.2	145	1.3	0	0	0	0	0	0
2018	7,543,825	12,426	1298	10.4	1,732	13.9	363	2.9	21	0.2	176	1.4	0	0	2	0	0	0
2019	7,619,494	13,304	1348	10.1	1,930	14.5	631	4.7	22	0.2	212	1.6	0	0	7	0.1	0	0
2020	7,722,203	13,880	1364	9.8	2,061	14.8	845	6.1	20	0.1	253	1.8	0	0	16	0.1	0	0
2021	7,739,758	14,527	1484	10.2	2,233	15.4	1,081	7.4	21	0.1	269	1.9	1	0.1	19	0.1	0	0
2022	7,758,615	15,133	1639	10.8	2,424	16	1,354	8.9	26	0.2	292	1.9	17	1.7	12	0.1	0	0
2023	8,016,606	15,694	1791	11.4	2,590	16.5	1,605	10.2	30	0.2	330	2.1	68	6.8	12	0.1	1	0.01

Supplementary Table S10. Biologic treatment in UC patients. Percentages are calculated by dividing the number of patients receiving the drug by the total number of patients with UC

	**Catalan Population**	**UC patients**	IFX		ADA		USTE		GOLI		VEDO		UPA		TOFA		FILGO	
			**Number**	**%**	**Number**	**%**	**Number**	**%**	**Number**	**%**	**Number**	**%**	**Number**	**%**	**Number**	**%**	**Number**	**%**
2017	7,496,276	17,534	597	3.4	296	1.7	17	0.1	111	0.6	130	0.7	0	0	2	0	0	0
2018	7,543,825	19,747	632	3.2	350	1.8	37	0.2	144	0.7	217	1.1	0	0	20	0.1	0	0
2019	7,619,494	21,262	703	3.3	403	1.9	77	0.4	154	0.7	292	1.4	0	0	55	0.3	0	0
2020	7,722,203	22,301	724	3.2	462	2.1	110	0.5	135	0.6	360	1.6	0	0	104	0.5	0	0
2021	7,739,758	23,531	796	3.4	561	2.4	177	0.8	140	0.6	430	1.8	6	0	131	0.6	0	0
2022	7,758,615	24,644	910	3.7	610	2.5	314	1.3	136	0.6	459	1.9	12	0	147	0.6	0	0
2023	8,016,606	25,728	1,074	4.2	691	2.7	396	1.5	131	0.5	501	1.9	36	0.1	173	0.7	18	0.1

Supplementary Table S11. Surgical and diagnostic procedures in IBD from 2017 to 2023. Absolute number and rate per 1000 patients/year.

	Population of Catalonia	Persons/year	Ostomies		Resections		Other surgical procedures	
			Cases	Rate x 1.000	Cases	Rate x 1.000	Cases	Rate x 1.000
2017	7,496,276	27,614	204	7.4	288	10.4	249	9.0
2018	7,543,825	30,349	202	6.7	312	10.3	240	7.9
2019	7,619,494	33,155	198	6.0	286	8.6	227	6.8
2020	7,722,203	34,967	231	6.6	301	8.6	239	6.8
2021	7,739,758	36,768	232	6.3	332	9.0	281	7.6
2022	7,758,615	38,527	229	5.9	341	8.9	162	4.2
2023	8,016,606	40,083	233	5.8	360	9.0	143	3.6

Supplementary Table S12. Surgical and diagnostic procedures in CD from 2017 to 2023. Absolute number and rate per 1000 patients/year.

	Population of Catalonia	CD patients	Ostomies		Resections		Other surgical procedures	
			n	%	n	%	n	%
2017	7,496,276	10,759	122	11.3	187	17.1	190	17.7
2018	7,543,825	11,780	104	8.8	165	14.0	164	13.9
2019	7,619,494	12,805	104	8.1	138	10.8	157	12.3
2020	7,722,203	13,454	95	7.1	143	10.6	157	11.7
2021	7,739,758	14,106	93	6.6	147	10.4	192	13.6
2022	7,758,615	14,717	99	6.7	157	10.7	91	6.2
2023	8,016,606	15,215	116	7.6	170	11.2	86	5.7

Supplementary Table S13. Surgical and diagnostic procedures in UC from 2017 to 2023. Absolute number and rate per 1000 patients/year.

	**Population of Catalonia**	**UC patients**	**Ostomies**		**Resections**		**Other surgical procedures**	
			**n**	**%**	**n**	**%**	**n**	**%**
2017	7,496,276	16,855	82	4.9	101	6.0	59	3.5
2018	7,543,825	18,570	98	5.3	147	7.9	76	4.1
2019	7,619,494	20,350	94	4.6	148	7.3	70	3.4
2020	7,722,203	21,513	136	6.3	158	7.3	82	3.8
2021	7,739,758	22,662	139	6.1	185	8.2	89	3.9
2022	7,758,615	23,810	130	5.5	184	7.7	71	3.0
2023	8,016,606	24,869	117	4.7	190	7.6	57	2.3

Supplementary Table S14. Hospitalization in IBD patients. Absolute number and rate per 1000 patients/year

	**Persons/year**	Total		IBD		Other GI disease		Neoplasia		Infections		Other autoimmune		Complications		Others	
		**n**	**Rate x1.000**	**n**	**Rate x1.000**	**n**	**Rate x1.000**	**n**	**Rate x1.000**	**n**	**Rate x1.000**	**n**	**Rate x1.000**	**n**	**Rate x1.000**	**n**	**Rate x1.000**
2017	27,614	7,147	258.8	951	34.4	1,319	47.8	490	17.7	281	10.2	35	1.3	218	7.9	3,911	141.6
2018	30,349	7,521	247.8	1,040	34.3	1,255	41.4	545	18	233	7.7	29	1	252	8.3	4,201	138.4
2019	33,155	8,253	248.9	1,143	34.5	1,413	42.6	613	18.5	304	9.2	45	1.4	308	9.3	4,483	135.2
2020	34,967	7,207	206.1	967	27.7	1,178	33.7	606	17.3	334	9.6	39	1.1	230	6.6	3,900	111.5
2021	36,768	8,558	232.8	1,064	28.9	1,319	35.9	784	21.3	241	6.6	32	0.9	302	8.2	4,856	132.1
2022	38,527	9,391	243.8	1,047	27.2	1,443	37.5	783	20.3	311	8.1	37	1	323	8.4	5,495	142.6
2023	40,083	10,121	252.5	1,096	27.3	1,532	38.2	838	20.9	357	8.9	35	0.9	410	10.2	5,890	146.9

Supplementary Table S15. Hospitalization in CD patients. Absolute number and rate per 1000 patients/year

	Persons/year	Total		IBD		Other GI disease		Neoplasia		Infections		Other autoimmune		Complications		Others	
		**n**	**Rate x1.000**	**n**	**Rate x1.000**	**n**	**Rate x1.000**	**n**	**Rate x1.000**	**n**	**Rate x1.000**	**n**	**Rate x1.000**	**n**	**Rate x1.000**	**n**	**Rate x1.000**
2017	10,759	3,155	293.2	598	55.6	690	64.1	170	15.8	129	12	14	1.3	104	9.7	1,478	137.4
2018	11,780	3,215	272.9	697	59.2	573	48.6	196	16.6	103	8.7	7	0.6	108	9.2	1,543	131
2019	12,805	3,527	275.4	712	55.6	666	52	242	18.9	128	10	8	0.6	116	9.1	1,666	130.1
2020	13,454	3,062	227.6	623	46.3	550	40.9	206	15.3	105	7.8	7	0.5	111	8.3	1,471	109.3
2021	14,106	3,488	247.3	652	46.2	592	42	285	20.2	95	6.7	8	0.6	111	7.9	1,757	124.6
2022	14,717	3,744	254.4	614	41.7	624	42.4	283	19.2	119	8.1	10	0.7	134	9.1	1,975	134.2
2023	15,215	3,989	262.2	678	44.6	667	43.8	254	16.7	150	9.9	13	0.9	159	10.5	2,082	136.8

Supplementary Table S16. Hospitalization in UC patients. Absolute number and rate per 1000 patients/year

	**Persons/year**	Total		IBD		Other GI disease		Neoplasia		Infections		Other autoimmune		Complications		Others	
		**n**	**Rate x1.000**	**n**	**Rate x1.000**	**n**	**Rate x1.000**	**n**	**Rate x1.000**	**n**	**Rate x1.000**	**n**	**Rate x1.000**	**n**	**Rate x1.000**	**n**	**Rate x1.000**
2017	16,855	3,992	236.8	353	20.9	629	37.3	320	19	152	9	21	1.2	114	6.8	2,433	144.3
2018	18,570	4,306	231.9	343	18.5	682	36.7	349	18.8	130	7	22	1.2	144	7.8	2,658	143.1
2019	20,350	4,726	232.2	431	21.2	747	36.7	371	18.2	176	8.6	37	1.8	192	9.4	2,817	138.4
2020	21,513	4,145	192.7	344	16	628	29.2	400	18.6	229	10.6	32	1.5	119	5.5	2,429	112.9
2021	22,662	5,070	223.7	412	18.2	727	32.1	499	22	146	6.4	24	1.1	191	8.4	3,099	136.7
2022	23,810	5,647	237.2	433	18.2	819	34.4	500	21	192	8.1	27	1.1	189	7.9	3,520	147.8
2023	24,869	6,132	246.6	418	16.8	865	34.8	584	23.5	207	8.3	22	0.9	251	10.1	3,808	153.1

Supplementary Table S17. STROBE Statement. Checklist of items that should be included in reports of cross-sectional studies

	**Item No**	**Recommendation**	**Page No**

**Title and abstract**	1	(*a*) Indicate the study's design with a commonly used term in the title or the abstract	1
		(*b*) Provide in the abstract an informative and balanced summary of what was done and what was found	1
**Introduction**			
Background/rationale	2	Explain the scientific background and rationale for the investigation being reported	2
Objectives	3	State specific objectives, including any prespecified hypotheses	2
**Methods**			
Study design	4	Present key elements of study design early in the paper	2
Setting	5	Describe the setting, locations, and relevant dates, including periods of recruitment, exposure, follow-up, and data collection	2
Participants	6	(*a*) Give the eligibility criteria, and the sources and methods of selection of participants	2
Variables	7	Clearly define all outcomes, exposures, predictors, potential confounders, and effect modifiers. Give diagnostic criteria, if applicable	2
Data sources/ measurement	8*	For each variable of interest, give sources of data and details of methods of assessment (measurement). Describe comparability of assessment methods if there is more than one group	2-3
Bias	9	Describe any efforts to address potential sources of bias	2-3
Study size	10	Explain how the study size was arrived at	2-3
Quantitative variables	11	Explain how quantitative variables were handled in the analyses. If applicable, describe which groupings were chosen and why	2-3
Statistical methods	12	(*a*) Describe all statistical methods, including those used to control for confounding	3
		(*b*) Describe any methods used to examine subgroups and interactions	N/A
		(*c*) Explain how missing data were addressed	N/A

		(*d*) If applicable, describe analytical methods taking account of sampling strategy	N/A
		(*e*) Describe any sensitivity analyses	N/A
**Results**			
Participants	13*	(a) Report numbers of individuals at each stage of study—eg numbers potentially eligible, examined for eligibility, confirmed eligible, included in the study, completing follow-up, and analysed	4
		(b) Give reasons for non-participation at each stage	N/A
		(c) Consider use of a flow diagram	N/A
Descriptive data	14*	(a) Give characteristics of study participants (eg demographic, clinical, social) and information on exposures and potential confounders	4
		(b) Indicate number of participants with missing data for each variable of interest	4
Outcome data	15*	Report numbers of outcome events or summary measures	4-7
Main results	16	(*a*) Give unadjusted estimates and, if applicable, confounder-adjusted estimates and their precision (eg, 95% confidence interval). Make clear which confounders were adjusted for and why they were included	6
		(*b*) Report category boundaries when continuous variables were categorized	N/A
		(*c*) If relevant, consider translating estimates of relative risk into absolute risk for a meaningful time period	6
Other analyses	17	Report other analyses done—eg analyses of subgroups and interactions, and sensitivity analyses	7
**Discussion**			
Key results	18	Summarise key results with reference to study objectives	7
Limitations	19	Discuss limitations of the study, taking into account sources of potential bias or imprecision. Discuss both direction and magnitude of any potential bias	9

Interpretation	20	Give a cautious overall interpretation of results considering objectives, limitations, multiplicity of analyses, results from similar studies, and other relevant evidence	9
Generalisability	21	Discuss the generalisability (external validity) of the study results	9
**Other information**			
Funding	22	Give the source of funding and the role of the funders for the present study and, if applicable, for the original study on which the present article is based	11

*Give information separately for exposed and unexposed groups.

Supplementary Figure S1: Sex and age distribution for CD (a) and UC (b) patients in Catalonia in 2023

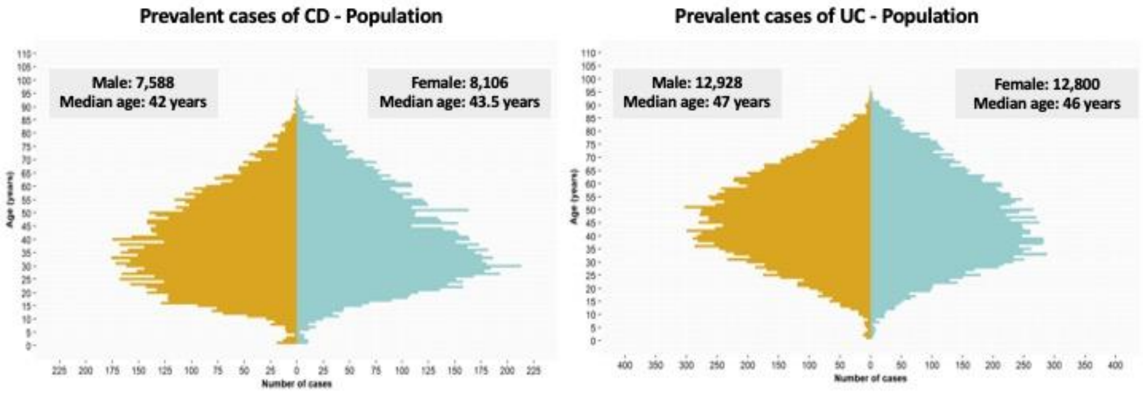

Supplementary Figure S2. Trends and evolution of mean ages of prevalent IBD cases in Catalonia between 2017 and 2023. Data for CD and UC.

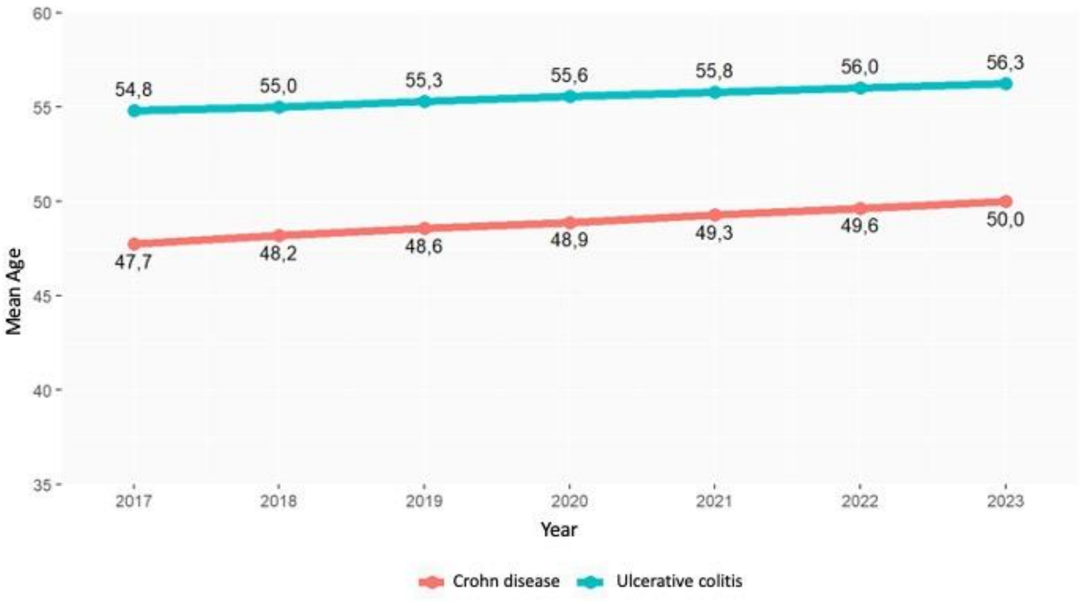

Supplementary Figure S3. Trends of use of IBD treatments in Catalonia between 2017 and 2023.

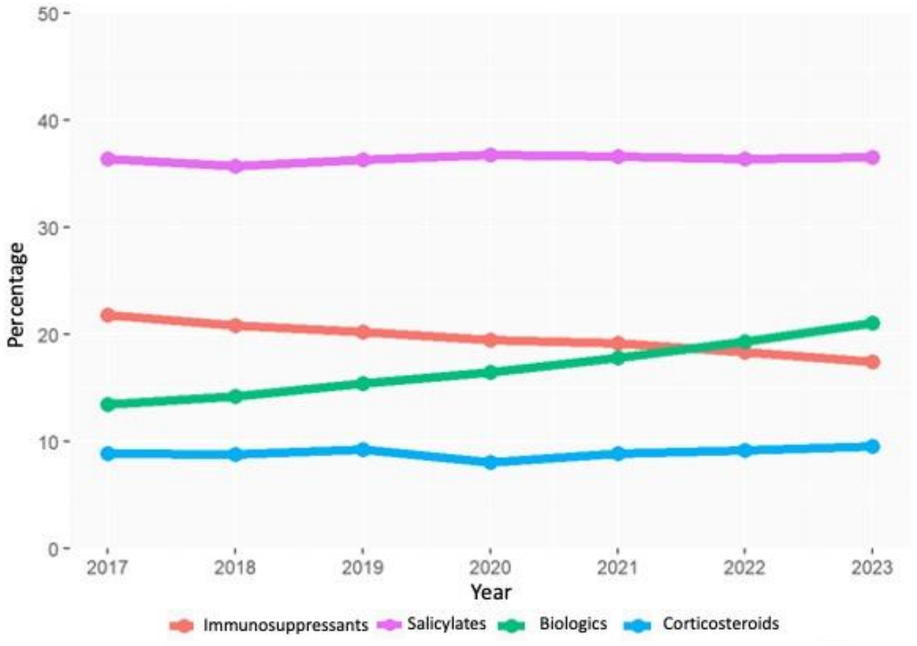

Supplementary Figure S4. Trends of use of biological and iJAKs therapies for IBD in Catalonia between 2017 and 2023.

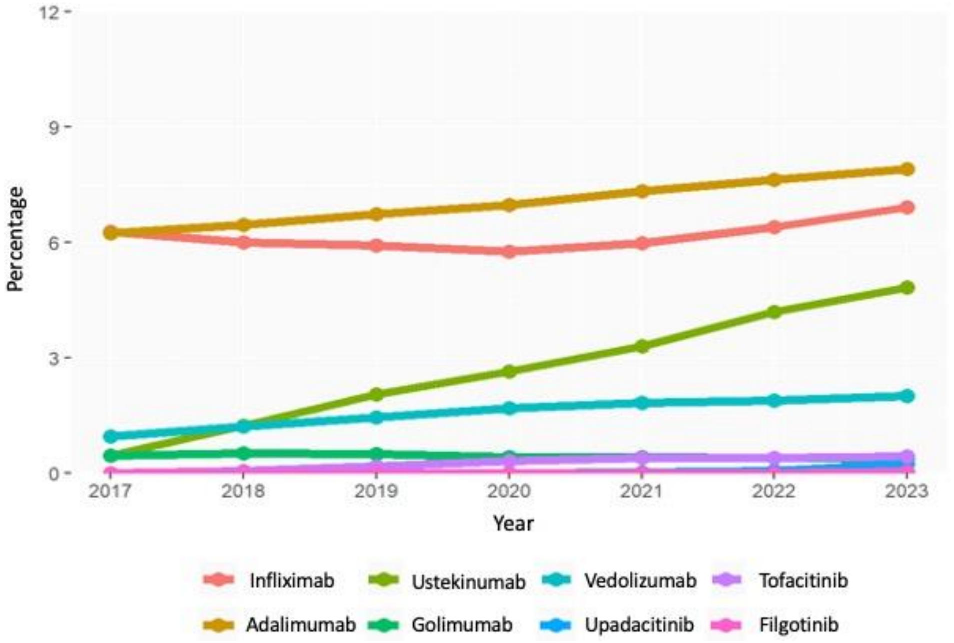

Supplementary Figure S5. Surgical trends for IBD patients in Catalonia between 2017 and 2023.

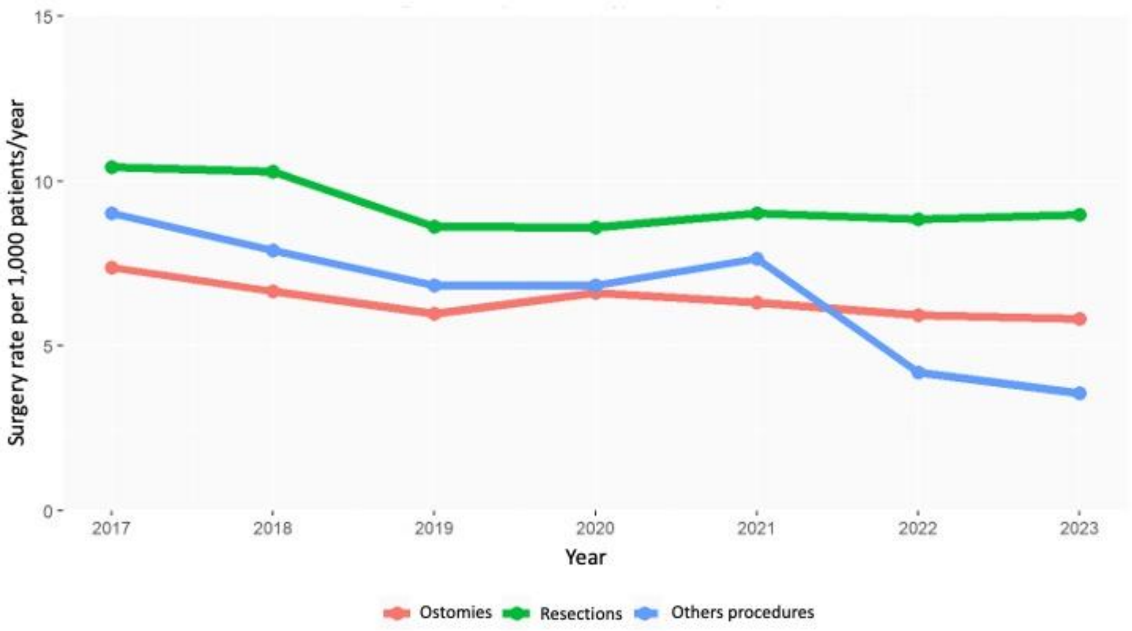

Supplementary Figure S6. Correlations between treatments for IBD (a), CD (b), and UC (c) and outcomes (surgeries and hospitalization). Only significant correlations are shown.

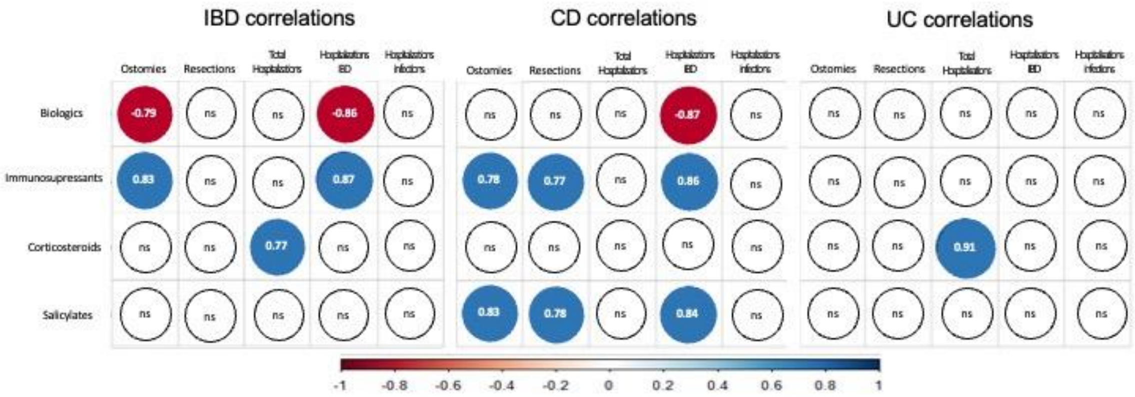

Supplementary Figure S7. Trends in hospitalization for IBD patients in Catalonia between 2017 and 2023.

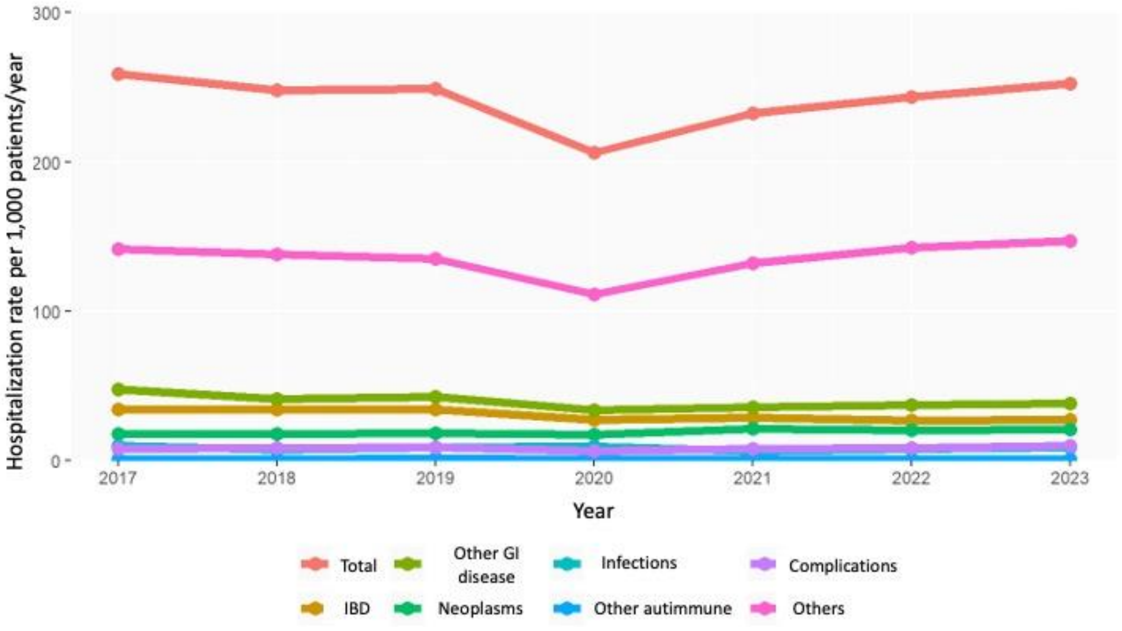

Supplement: Supplementary file 1 [file jcm-14-05711-s001.zip › jcm-3794834-supplementary.pdf]
